# Identification and validation of prognosis‐related *DLX5* methylation as an epigenetic driver in myeloid neoplasms

**DOI:** 10.1002/ctm2.29

**Published:** 2020-06-04

**Authors:** Ting‐juan Zhang, Zi‐jun Xu, Yu Gu, Xiang‐mei Wen, Ji‐chun Ma, Wei Zhang, Zhao‐qun Deng, Jia‐yan Leng, Jun Qian, Jiang Lin, Jing‐dong Zhou

**Affiliations:** ^1^ Department of Hematology Affiliated People's Hospital of Jiangsu University Zhenjiang P. R. China; ^2^ Zhenjiang Clinical Research Center of Hematology Zhenjiang P. R. China; ^3^ The Key Lab of Precision Diagnosis and Treatment in Hematologic Neoplasms of Zhenjiang City Zhenjiang P. R. China; ^4^ Laboratory Center Affiliated People's Hospital of Jiangsu University Zhenjiang P. R. China

**Keywords:** AML, *DLX5*, MDS, methylation, progression

## Abstract

The deregulated *DLX* gene family members *DLX1/2/3/4/5/6* (*DLXs*) caused by DNA methylation has been demonstrated in various cancers with therapeutic target value. However, the potential role of *DLXs* methylation in myeloid neoplasms such as acute myeloid leukemia (AML) and myelodysplastic syndromes (MDS) remains to be elucidated. Clinical significance of *DLXs* methylation/expression was analyzed in patient with AML and MDS. The functional roles of *DLXs* were determined in vitro. In the identification stage, we found that lower *DLX5* expression was correlated with prognosis in AML among all *DLXs* analyzed by The Cancer Genome Atlas datasets. In the validation stage, we revealed that reduced *DLX5* expression was frequently occurred, and was also correlated with promoter hypermethylation in AML evaluated by targeted bisulfite sequencing. Epigenetic studies also showed that *DLX5* promoter DNA methylation was associated with its expression. By quantitative polymerase chain reaction, we also validated that *DLX5* hypermethylation was frequent event in both AML and MDS, and also correlated with MDS transformation to leukemia. Moreover, *DLX5* hypermethylation was associated with lower rate of complete remission and shorter time of leukemia‐free/overall survival, and was also confirmed by Logistic/Cox regression analysis. Functional studies revealed the antiproliferative and pro‐apoptotic effects of *DLX5* in MDS‐derived AML cell‐line SKM‐1. Finally, bioinformatics analysis demonstrated that *DLX5* functioned in leukemogenesis may be through the association with PI3K/Akt signaling pathway. Collectively, our findings demonstrated that *DLX5* methylation, negatively correlated *DLX5* expression, was a potential prognostic and predictive indicator in patients with AML and MDS, which could also act as an epigenetic driver in myeloid neoplasms.

**Abbreviations**
5‐aza‐dC: 5‐aza‐2′‐deoxycytidine
AML: acute myeloid leukemia
AUC: area under the ROC curve
BM: bone marrow
BMMNCs: BM mononuclear cells
CN‐AML: cytogenetically normal AML
CR: complete remission
*DLXs*: *DLX* gene familymembers (*DLX1/2/3/4/5/6* *)*
FAB: French‐American‐British
LFS: leukemia‐free survival
MDS: myelodysplastic syndromes
OS: overall survival
ROC: receiver operating characteristic
RT‐qMSP: real‐time quantitative methylation‐specific PCR
RT‐qPCR: real‐time quantitative polymerase chain reaction
TCGA: The Cancer Genome Atlas

## 1 INTRODUCTION

Acute myeloid leukemia (AML) and myelodysplastic syndromes (MDS) are clonal diseases of myeloid hematopoietic stem cells, and seen as the most common aggressive diseases of myeloid neoplasms.^1^, ^2^ AML is characterized by normal hematopoietic failure caused by clonal proliferation of unconscionable blasts,^1^ whereas MDS is defined as invalid hematopoiesis and high risk of progression into AML, also regarded as a preleukemia disease.^2^ Both AML and MDS are clinically and biologically heterogeneous disorders due to the genetically and molecular diverse.^1^, ^2^ In spite of the recent advances made in the personalized and precision medicine, the outcome of AML and MDS remains poor particularly in high‐risk MDS and AML including the MDS‐derived AML.^1^, ^3^ Consequently, the identification of molecular alterations as potential biomarkers associated with prognosis and progression in MDS and AML could give better insights into leukemogenesis that allow molecular‐based therapies, and finally may improve clinical outcome of AML and MDS.

The *DLX* gene family members *DLX1/2/3/4/5/6*(*DLXs*), which correlated with Drosophila distal‐less gene, belongs to the homeobox gene subfamily. *DLXs* encode transcription factors that are expressed in the development of embryonic including appendages, nervous system, branchial arches, and hematopoiesis by regulating cell growth and differentiation.^4^, ^5^ To date, a total of six *DLXs* are identified, represented by three clusters, namely, *DLX1*/*DLX2*, *DLX3*/*DLX4*, and *DLX5*/*DLX6*. Accumulating studies have showed the deregulated *DLXs* caused by DNA methylation in various cancers with therapeutic target value.^6^, ^7^ For example, Tong et al revealed that *DLX1* and *DLX4* were frequently hypermethylated, which played a role in silencing gene expression by whole‐genome wide methylation analysis and pyrosequencing in chronic lymphocytic leukemia.^8^ The locus‐specific hypermethylation of oncogenic homeobox gene *DLX1* gene‐body canyon can upregulate its gene expression by pan‐cancer analysis.^9^ The homeobox genes (*IRX2*, *DLX2*, and *NKX2‐2*) hypermethylation was identified correlated with Luminal A subtype in breast cancer.^10^ Additionally, both *DLX1* and *DLX2* CpG island hypermethylation were shown in human astrocytomas.^11^ As for *DLX3*/*DLX4*, studies have reported that reduced *DLX3* expression was mediated by DNA hypermethylation at its promoter region in MLL‐AF4 childhood acute lymphoblastic leukemia.^12^ Notably, our previous studies have disclosed that *DLX4* hypermethylation was frequently occurred in myeloid neoplasms including AML, MDS as well as chronic myeloid leukemia, and predicted unfavorable prognosis or disease progression.^13^, ^14^, ^15^ In the cluster *DLX5*/*DLX6*, the phenomena of *DLX5* hypermethylation was identified in breast cancer, neuroblastoma tumors, and colorectal cancer.^16^, ^17^, ^18^ Until now, the potential role of *DLXs* methylation in myeloid neoplasms remains to be elucidated.

In this investigation, (a) we first identified and validated that *DLX5* decreased expression, of all the *DLXs*, was correlated with prognosis in AML, and may be caused by promoter hypermethylation. (b) Next, by the targeted bisulfite sequencing and real‐time quantitative methylation‐specific polymerase chain reaction (RT‐qMSP), we further validated and confirmed that *DLX5* hypermethylation was frequently occurred in AML and MDS, and observed that *DLX5* methylation was associated with leukemia transformation in MDS. Moreover, *DLX5* methylation predicted unfavorable prognosis in both MDS and AML. (c) Functional studies demonstrated the antiproliferative and pro‐apoptotic effects of *DLX5* in MDS‐derived AML cell‐line SKM‐1. (d) Finally, bioinformatics analysis revealed that *DLX5* functioned in leukemogenesis may be through the association with PI3K/Akt signaling pathway.

## 2 MATERIALS AND METHODS

### 2.1 Cases and samples

The investigation was approved by the Ethics Committee of Affiliated People's Hospital of Jiangsu University. First, we analyzed 200 AML patients (173 cases with *DLXs* expression) downloaded from The Cancer Genome Atlas (TCGA) databases from the Washington University as reported.^19^ Second, we analyzed two cohorts of AML and MDS patients as well as controls from our hospital. The first cohort included 111 de novo AML patients, 35 MDS patients, and 25 healthy volunteers, which was used for targeted bisulfite sequencing. The other cohort enrolled 159 AML cases (148 de novo AML and 11 MDS‐derived AML), 61 MDS patients, and 46 healthy volunteers, which was used for RT‐qMSP. Patients with antecedent hematological diseases (except for MDS‐derived AML) or solid tumors or therapy‐related diseases were excluded. The diagnosis of AML and MDS was based on the 2008 revised World Health Organization (WHO) criteria and the French‐American‐British (FAB) classifications.^20^ The sex and age distribution in case and control group showed no statistical significance. The treatment regimens of these patients were as our previous work.^21^, ^22^, ^23^ Common gene mutations of these patients were detected previously.^21^, ^22^, ^23^ Bone marrow (BM) specimens were obtained from all participants at diagnosis time after signing informed consents. Total RNA specimens were extracted from BM mononuclear cells (BMMNCs), separating from BM samples by Lymphocyte Separation Medium (Solarbio, Beijing, China), using TRIzol reagent. Genomic DNA samples were collected from BMMNCs by Puregene Blood Core Kit B (QIAGEN, Duesseldorf, Germany).

### 2.2 Targeted bisulfite sequencing

Targeted bisulfite sequencing (called as MethylTarget) was performed to detect *DLX5* methylation density (Genesky Biotechnologies Inc., Shanghai, China). The primers used for *DLX5* are shown in Table S1. A detailed description of the targeted bisulfite sequencing assay was reported previously.^24^

### 2.3 Reverse transcription and real‐time quantitative polymerase chain reaction

Reverse transcription was performed using random primers.^21^, ^22^, ^23^ The program of RT was conducted based on the manufacturer's instructions. Determination of *DLX5* mRNA expression level was evaluated by real‐time quantitative polymerase chain reaction (RT‐qPCR) using AceQ qPCR SYBR Green Master Mix (Vazyme, Piscataway, NJ). The program of RT‐qPCR was performed on a Thermo cycler (ABI 7500, Foster City, CA, USA). Both positive controls (recombinant plasmid) and negative controls (ddH_2_O) were added in each PCR reaction. *ABL1* was applied to evaluate relative *DLX5* mRNA expression. The primers used for *DLX5* are shown in Table S1. The relative expression of *DLX5* mRNA was calculated by 2^–∆∆^ *^CT^* method.^23^

### 2.4 Bisulfite modification and RT‐qMSP

Genomic DNA was bisulfite converted as reported.^21^, ^22^, ^23^ The *DLX5* methylation level was detected by RT‐qMSP using the same agents as described in RT‐qPCR with primers listed in Table S1. Both positive controls (recombinant plasmid) and negative controls (ddH_2_O) were added in each PCR reaction. *ALU* was also detected to assess the relative level of *DLX5* methylation. Relative methylation level of *DLX5* was calculated by 2^–∆∆^ *^CT^* method.^23^

### 2.5 Cell‐line and culture

The MDS‐derived AML cell‐line SKM‐1 were cultured in 10% fetal calf serum of RPMI 1640 medium, which was grown at 37°C in 5% CO_2_ humidified atmosphere.^25^, ^26^

### 2.6 Demethylation studies

SKM‐1 cells in 2 mL (5 × 10^5^ cells/mL) were treated with 5‐aza‐2′‐deoxycytidine (5‐aza‐dC) with a final concentration of 10 µM during 4 days.

### 2.7 Cell transfection

Human full‐length *DLX5* coding sequence was introduced into the BamHI/AgeI of GV569 vector (GENECHEM, Shanghai, China), and transfected with lentiviruses.

### 2.8 Western blot analysis

The procedures of western blotting were conducted as described.^23^, ^27^ The antibodies included in this investigation were rabbit anti‐*DLX5* (Abcam, Cambridge, MA, USA) and mouse anti‐GAPDH (BOSTER, Wuhan, China).

### 2.9 Cell growth assays

Cells were distributed equally to at 96‐well plates (5 × 10^3^ per well). After culture for 0, 1, 2, 3, 4, and 5 days, CCK‐8 (Dojindo, Kumamoto, Japan) reagent was used to analyze the cell proliferation. The rate of cell growth was calculated as OD (Optical Delnsity) value, which was measured at 450 nm of the absorbance using a microplate reader.

### 2.10 Cell apoptosis analysis

Cells were distributed equally to at 96‐well plates (5 × 10^5^ per well). After culture for 2 days, Annexin V‐APC Kit was applied to analyze the apoptosis rate based on the recommended protocols, and detected by flow cytometry.

### 2.11 Cell cycle analysis

Cells were distributed equally to at 96‐well plates (5 × 10^4^ per well). After culture for 2 days, PI/RNase staining buffer Kit was applied to analyze the cell cycle distribution based on the recommended protocols, and detected by flow cytometry.

### 2.12 Bioinformatics analysis

The details regarding the bioinformatics analysis of *DLX5* were as reported previously.^28^

### 2.13 Statistical analysis

SPSS software 20.0 and GraphPad Prism 5.0 were used in statistical analysis. The difference of continuous variables in two groups was compared by Mann‐Whitney's *U*‐test/Student *T*‐test, whereas Pearson Chi‐square analysis/Fisher exact test was applied to analyze the difference of categorical variables in two groups. The correlation analysis between *DLX5* methylation and expression or *DLX5* methylation density and methylation level was analyzed by Spearman test. The distinguishing value of *DLX5* methylation between AML and controls was analyzed by receiver operating characteristic (ROC) curve and area under the ROC curve (AUC). Complete remission (CR) was evaluated after one or two course of induction chemotherapy. Overall survival (OS) and leukemia‐free survival (LFS) of AML and MDS patients were defined as our previous report.^21^ Kaplan‐Meier analysis and Cox regression analysis (including univariate and multivariate analysis) were applied to evaluate the prognostic effect of *DLX5* methylation on OS and LFS. The definition of statistical significance was attached when a two‐sided *P *< .05.

## 3 RESULTS

### 3.1 Identification of DLXs expression associated with prognosis in AML by public database

**FIGURE 1:**
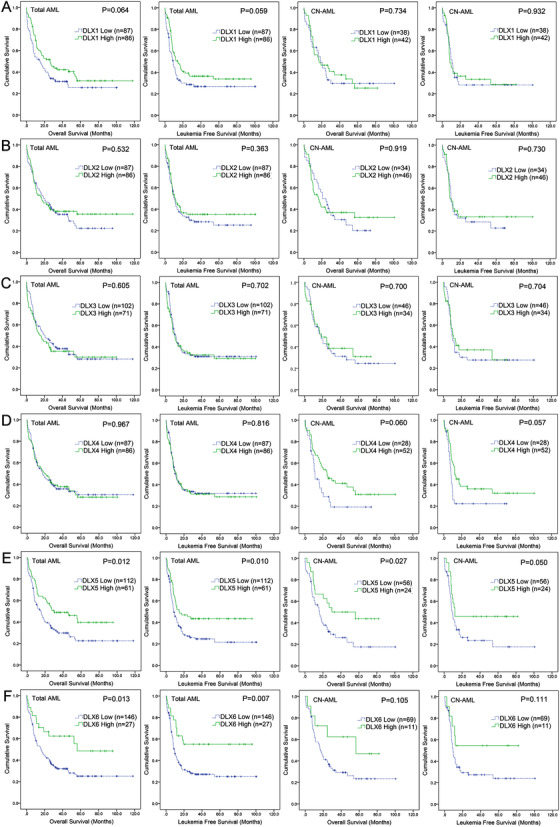
Identification of *DLXs* expression associated with prognosis in AML by public database. A‐F, *DLX1‐6*. The impact of *DLXs* expression on overall survival and leukemia‐free survival was determined among whole‐cohort AML and cytogenetically normal AML patients from TCGA databases. AML patients were divided into two groups by the median methylation level of each gene, respectively

We first used the public TCGA data to identify the prognosis‐related genes of *DLXs* (*DLX1*/*2*/*3*/*4*/*5*/*6*) in AML. Prognostic significance of all *DLXs* was analyzed in two groups divided by the median level of each gene expression (*DLX1*/*2*/*3*/*4*/*5*/*6*), respectively. Kaplan‐Meier analysis revealed that only *DLX5* expression was positively correlated with OS and LFS in both whole‐cohort AML (*P *= .012 and .010; Figure 1) and cytogenetically normal AML (CN‐AML) patients (*P *= .027 and .050; Figure 1), suggesting the negatively prognostic value of *DLX5* expression in AML.

### 3.2 Validation of DLX5 under‐expression in AML associated with promoter methylation

**FIGURE 2:**
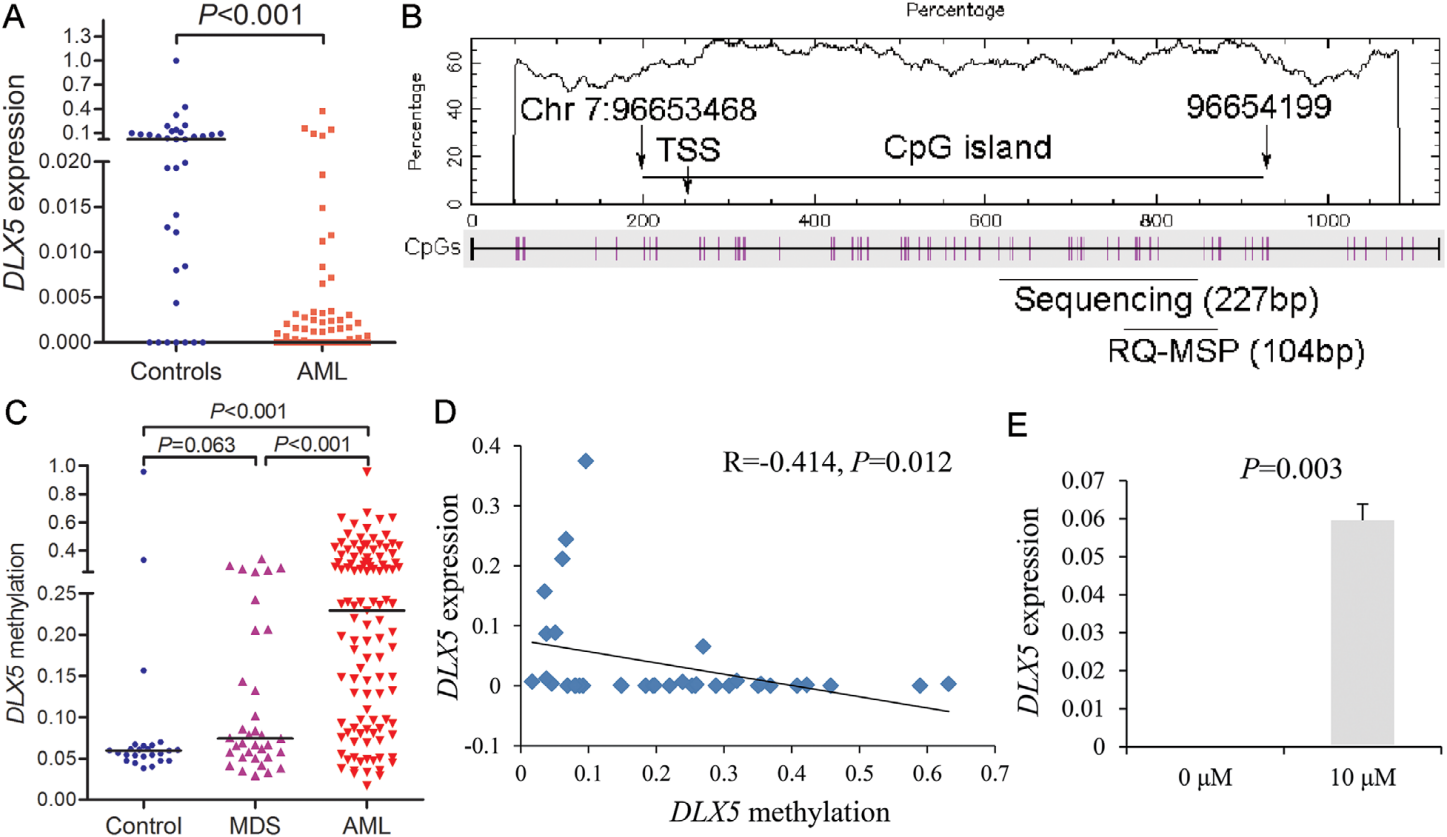
Validation of *DLX5* under‐expression in AML associated with promoter methylation. A, *DLX5* expression level in controls and AML patients examined by real‐time quantitative polymerase chain reaction (RT‐qPCR). B, The genomic coordinates (GC) of *DLX5* promoter region CpG island and primer locations. The panel plots the GC content as a percentage of the total. Each vertical bar in the bottom panel represents the presence of a CpG dinucleotide. Black horizontal lines indicate regions amplified by sequencing primer pairs and real‐time quantitative methylation‐specific polymerase chain reaction (RT‐qMSP) primer pairs. CpGplot (http://emboss.bioinformatics.nl/cgi-bin/emboss/cpgplot) and Methyl Primer Express v1.0 software were used for creating the figure (TSS, transcription start site). C, *DLX5* methylation density in controls, MDS, and AML patients detected by targeted bisulfite sequencing. D, Correlation between *DLX5* methylation (targeted bisulfite sequencing) and expression (RT‐qPCR) in MDS and AML patients. The correlation analysis was conducted by Spearman test. E, *DLX5* expression in MDS‐derived AML cell line SKM‐1 before and after 10‐µM 5‐aza‐dC treatment

To validate the expression pattern of *DLX5* in AML, we first performed RT‐qPCR to detect *DLX5* mRNA level in 86 de novo AML patients and 35 controls. As showed in Figure 2A, *DLX5* expression was markedly decreased in de novo AML patients (*P *< .001). As reported previously, dysregulation of *DLXs* including *DLX5* caused by DNA methylation in various cancers potentially served as therapeutic targets. We further detected CpG island methylation pattern located at the *DLX5* promoter region (Figure 2B) by targeted bisulfite sequencing in samples of 111 de novo AML patients, 35 MDS patients, and 25 controls. The sequencing mean bait coverage attached 1694×, and Q30 was 75.56%. Although no statistical significance was showed of *DLX5* methylation in MDS patients and controls (*P *= .063; Figure 2C), the level of *DLX5* methylation in AML patients was markedly higher than in controls and MDS patients (both *P *< .001; Figure 2C). Moreover, *DLX5* methylation was observed to be slightly negatively correlated with *DLX5* expression (*R* = –.414, *P *= .012; Figure 2D). In order to confirm the epigenetic mechanism, MDS‐derived AML cell line SKM‐1 were treated with demethylation agent 5‐aza‐dC. As a result, the level *DLX5* mRNA was markedly upregulated after 5‐aza‐dC treatment (*P *= .003; Figure 2E).

### 3.3 Further confirmation of DLX5 methylation by quantitative PCR in AML and MDS

**FIGURE 3:**
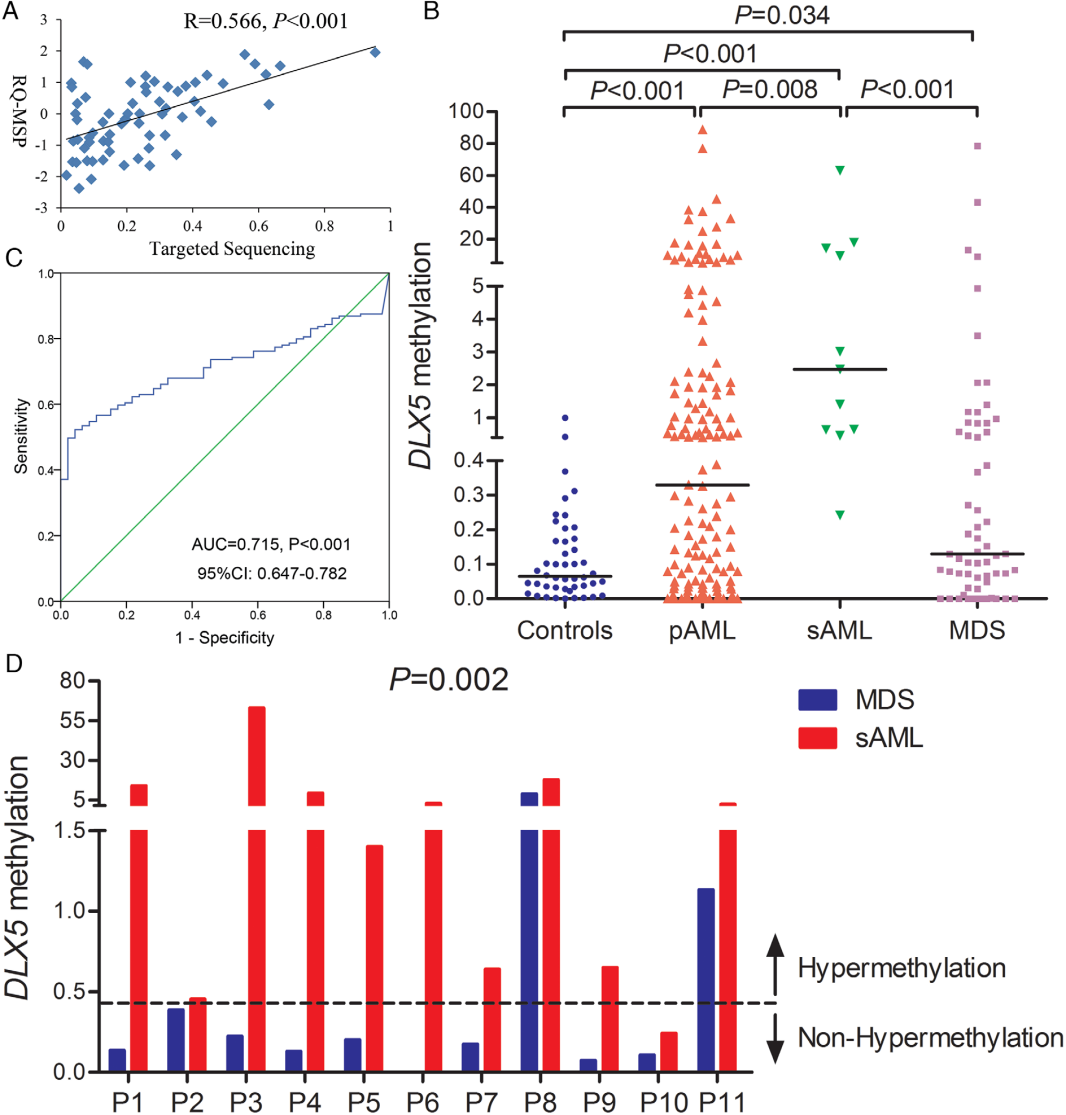
Further confirmation of *DLX5* methylation in AML and MDS patients together with its clinical significance. A, The correlation of the candidate gene methylation results between the targeted bisulfite sequencing and real‐time quantitative methylation‐specific polymerase chain reaction (RT‐qMSP). The correlation was analyzed by Spearman correlation test. B, *DLX5* methylation level in larger samples of controls, MDS, pAML, and sAML patients analyzed by RT‐qMSP. C, ROC curve analysis using *DLX5* methylation for discriminating AML patients from controls. D, *DLX5* methylation alterations in matched MDS/sAML patients examined by RT‐qMSP (pAML, primary AML; sAML, secondary AML, indicated MDS‐derived AML patients)

We further expanded the clinical samples (46 controls, 148 primary AML [pAML] and 11 secondary AML [sAML]) to explore clinical implication of *DLX5* methylation using a more rapid and convenient methodology RT‐qMSP. The primer was designed located inside the sequencing primer (Figure 2B), and RT‐qMSP results were positively associated with the results in targeted bisulfite sequencing (*R* = .566, *P *< .001; Figure 3A). According to RT‐qMSP, *DLX5* promoter was markedly hypermethylated in MDS, pAML, and sAML patients (*P *= .034, <.001, and <.001; Figure 3B). Interestingly, sAML patients also showed markedly higher methylation level of *DLX5* than pAML and MDS patients (*P *= .008 and <.001; Figure 3B). In addition, *DLX5* methylation may act as a potential marker for distinguishing AML from controls with an AUC of 0.715 analyzed by ROC analysis (95% CI, 0.647‐0.782, *P *< .001; Figure 3C). Moreover, the sensitivity was 49.7% and the specificity was 97.8% when *DLX5* methylation set at the cutoff value of 0.425. To analyze the clinical implications of *DLX5* methylation, we divided the MDS and AML patients into two groups (*DLX5* hypermethylation and *DLX5* nonhypermethylation) according to the set cutoff value, respectively. For AML, no significant differences were observed between two groups regarding age, WBC (white blood cells), HB (hemoglobin), PLT (platelets), as well as FAB and karyotype classifications (*P *> .05; Table 1). However, *DLX5* hypermethylation was associated with male patients (*P *= .022; Table 1). Among gene mutations, *DLX5* hypermethylation was associated *CEBPA* and *c‐KIT* mutations (*P *= .001 and .058; Table 1). There were no association of *DLX5* hypermethylation with other gene mutations (*P *> .05; Table 1). For MDS patients, none of the clinical characteristics were found to be associated *DLX5* hypermethylation (*P *> .05; Table 2).

**TABLE 1:** Comparison of clinical and laboratory features between *DLX5* hypermethylated and nonhypermethylated AML patients

Patient's features	Nonhypermethylated (n = 80)	Hypermethylated (n = 79)	*P*‐value
Sex, male/female	42/38	56/23	.022
Median age, years (range)	54 (18‐85)	57 (18‐86)	.367
Median WBC, ×10^9^/L (range)	9.65 (0.3‐528.0)	18.15 (0.8‐185.4)	.325
Median hemoglobin, g/L (range)	79 (40‐144)	77 (32‐135)	.803
Median platelets, ×10^9^/L (range)	43 (7‐264)	33 (3‐447)	.091
Median BM blasts, % (range)	53.5 (1‐97.5)	50.5 (6.5‐99)	.508
FAB classifications			.139
M0	1	1	
M1	4	4	
M2	27	35	
M3	21	9	
M4	12	18	
M5	12	7	
M6	3	3	
No data	0	2	
Karyotypes			.066
Normal	34	33	
t(8;21)	3	8	
inv(16)	1	0	
t(15;17)	20	8	
+8	2	3	
‐5/5q‐	0	0	
‐7/7q‐	0	1	
t(9;22)	1	1	
11q23	0	2	
Complex	10	7	
others	7	8	
No data	2	8	
Gene mutations			
*CEBPA* (+/–)	0/64	10/51	.001
*NPM1* (+/–)	7/57	4/57	.531
*FLT3*‐ITD (+/–)	6/58	5/56	1.000
*C‐KIT* (+/–)	1/63	6/55	.058
*N/K‐RAS* (+/–)	3/61	8/53	.121
*IDH1/2* (+/–)	3/61	0/61	.244
*DNMT3A* (+/–)	3/61	4/57	.713
*U2AF1* (+/–)	1/63	2/59	.613
*SRSF2* (+/–)	2/62	2/59	1.000
*SETBP1* (+/–)	1/63	1/60	1.000
CR, total AML (+/–)	41/32	26/42	.043
CR, non‐M3 AML (+/–)	27/30	20/40	.135
CR, CN‐AML (+/–)	19/14	10/19	.081

*Note*. Patients’ blasts less than 20% with t(15;17) cytogenetic aberrations.

Abbreviations: BM, bone marrow; CR, complete remission; FAB, French‐American‐British classification; WBC, white blood cells.

**TABLE 2:** Comparison of clinical and laboratory features between *DLX5* hypermethylated and nonhypermethylated MDS patients

Patient's features	Nonhypermethylated (n = 50)	Hypermethylated (n = 11)	*P*‐value
Sex (male/female)	26/24	8/3	.317
Median age, years (range)	59 (27‐86)	58 (31‐78)	.620
Median WBC, ×10^9^/L (range)	2.8 (0.6‐82.4)	2.5 (0.7‐7.4)	.699
Median hemoglobin, g/L (range)	62 (35‐140)	61 (40‐107)	.656
Median platelets, ×10^9^/L (range)	61 (0‐505)	79 (20‐654)	.240
Median BM blasts, % (range)	5 (0‐19)	11 (0‐18)	.159
WHO classifications			.258
RCUD/RARS	5	2	
RCMD/RCMD‐RS	19	1	
RAEB‐1	9	2	
RAEB‐2	15	6	
MDS with isolated del(5q)	2	0	
MDS‐U	0	0	
IPSS scores			.371
Low	6	0	
Int‐1	24	5	
Int‐2	9	1	
High	6	4	
No data	5	1	
Gene mutations			
*CEBPA* (+/–)	2/40	0/11	1.000
*IDH1/2* (+/–)	1/41	0/11	1.000
*DNMT3A* (+/–)	1/41	0/11	1.000
*U2AF1* (+/–)	4/38	1/10	1.000
*SRSF2* (+/–)	1/41	1/10	.375
*SF3B1* (+/–)	3/39	0/11	1.000

Abbreviations: BM, bone marrow; IPSS, International Prognostic Scoring System; MDS, myelodysplastic syndromes; WBC, white blood cells; WHO, World Health Organization.

### 3.4 *DLX5* methylation correlated with leukemia transformation in MDS

As presented above, sAML patients also showed markedly higher methylation level of *DLX5* than pAML and MDS patients (*P *= .008 and <.001; Figure 3B). We deduced that *DLX5* methylation may be correlated with disease evolution in MDS. To verify the hypothesis, we further detected *DLX5* methylation in 11 matched patients during evolution from MDS to AML (see patients’ details in Table S2). As expected, the level of *DLX5* methylation was markedly upregulated at sAML stage than that at MDS stage in all matched patients (*P *= .002; Figure 3D).

### 3.5 DLX5 methylation was associated with prognosis in MDS and AML

**FIGURE 4:**
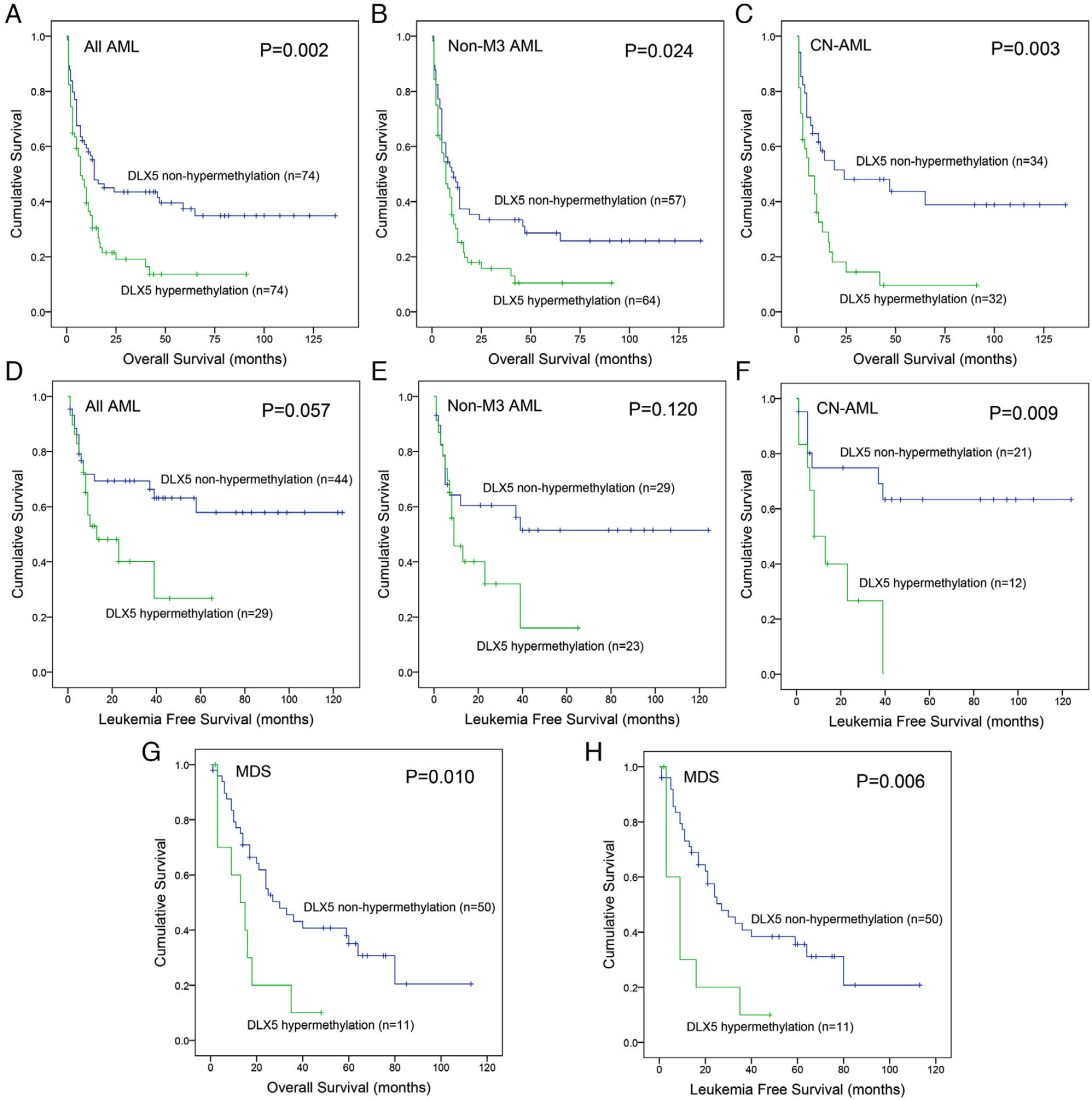
Prognostic value of *DLX5* methylation in AML and MDS patients. A‐C, The impact of *DLX5* methylation on overall survival among whole‐cohort AML, non‐M3‐AML, and cytogenetically normal AML (CN‐AML) patients, respectively. D‐F, The impact of *DLX5* methylation on leukemia‐free survival among whole‐cohort AML, non‐M3‐AML, and CN‐AML patients, respectively. G‐H, The impact of *DLX5* methylation on overall survival and leukemia‐free survival among MDS patients

For AML patients, we first observed the significant association of *DLX5* methylation with the rate of CR. Among whole‐cohort AML, the CR rate in *DLX5* hypermethylated patients was markedly lower than that in *DLX5* nonhypermethylated patients (*P *= .043; Table 1). In non‐M3 AML and CN‐AML, we did not observe the differences for CR between *DLX5* hypermethylated and nonhypermethylated patients (*P *= .135 and .081; Table 1). In addition, Logistic regression analysis including variables presented in Table S3 revealed that *DLX5* hypermethylation may be acted as a negative risk factor independently affecting CR in whole‐cohort AML (*P *= .067; Table S3). We next analyzed the prognostic value of *DLX5* methylation on AML survival (including LFS and OS). Kaplan‐Meier analysis demonstrated that *DLX5* hypermethylated cases had shorter OS time than *DLX5* nonhypermethylated cases among total AML, non‐M3 AML, and CN‐AML patients (*P *= .002, .024, and .003; Figure 4A‐C). For LFS, due the limited cases, significant difference was only observed in CN‐AML but not in total AML and non‐M3 AML between two groups (*P *= .009, .057, and .120; Figure 4D‐F). Furthermore, Cox regression analysis including variables presented in Tables 3 and S4 demonstrated that *DLX5* hypermethylation was a negatively prognostic factor independently affecting OS among whole‐chort AML with a trend (*P *= .071; Table S4) and CN‐AML patients (*P *= .025; Table 3).

**TABLE 3:** Cox regression analyses of variables for overall survival in CN‐AML patients

	Univariate analysis		Multivariate analysis	
Variables	Hazard ratio (95% CI)	*P*‐value	Hazard ratio (95% CI)	*P*‐value
*DLX5* methylation	2.389 (1.306‐4.369)	.005	2.345 (1.113‐4.942)	.025
Age	2.430 (1.340‐4.408)	.003	2.601 (1.283‐5.352)	.008
WBC	1.682 (0.930‐3.041)	.085	1.878 (0.888‐3.971)	.099
*CEBPA* mutations	6.498 (1.392‐30.329)	.017	5.949 (1.132‐31.279)	.035
*NPM1* mutations	0.375 (0.114‐1.230)	.105	0.385 (0.082‐1.812)	.227
*FLT3*‐ITD mutations	0.664 (0.234‐1.884)	.441		
*C‐KIT* mutations	0.044 (0.000‐15.404)	.296		
*N/K‐RAS* mutations	1.136 (0.398‐3.241)	.811		
*DNMT3A* mutations	0.973 (0.342‐2.768)	.960		
*U2AF1* mutations	2.797 (0.642‐12.191)	.171	4.489 (0.935‐21.559)	.061
*IDH1/2* mutations	0.693 (0.094‐5.095)	.719		
*SRSF2* mutations	4.501 (0.995‐20.367)	.051	3.105 (0.598‐16.120)	.178
*SETBP1* mutations	8.208 (0.988‐68.185)	.067	21.059 (2.179‐203.556)	.008

*Note*. Variables including *DLX5* methylation (hypermethylation vs nonhypermethylation), age (≤60 vs > 60 years), WBC (≥30×10^9^ vs < 30×10^9^ /L), and gene mutations (mutant vs wild type). Multivariate analysis includes variables with *P *< .200 in univariate analysis.

For MDS patients, we analyzed the prognostic value of *DLX5* methylation on MDS survival (also including OS and LFS). Kaplan‐Meier analysis revealed that *DLX5* hypermethylated patients presented markedly shorter OS and LFS than *DLX5* nonhypermethylated patients (*P *= .010 and .006; Figures 4G and 4H). In addition, Cox regression analysis including variables presented in Table 4 showed that *DLX5* hypermethylation also acted as an independently prognostically poor indicator for OS and LFS in MDS patients (*P *= .038 and .030; Table 4).

**TABLE 4:** Cox regression analyses of variables for overall survival and leukemia‐free survival in MDS patients

	Univariate analyses		Multivariate analyses	
Variables	Hazard ratio (95% CI)	*P*‐value	Hazard ratio (95% CI)	*P*‐value
Overall survival				
*DLX5* methylation	2.602 (1.211‐5.589)	.014	2.340 (1.050‐5.217)	.038
Age	1.888 (1.000‐3.564)	.050	1.862 (0.948‐3.658)	.071
IPSS risks	1.523 (1.063‐2.181)	.022	1.369 (0.949‐1.976)	.093
*CEBPA* mutation	0.044 (0.000‐14.199)	.289		
*IDH1/2* mutation	1.416 (0.192‐10.469)	.733		
*DNMT3A* mutation	1.696 (0.228‐12.601)	.606		
*U2AF1* mutation	1.107 (0.389‐3.153)	.849		
*SF3B1* mutation	0.602 (0.082‐4.424)	.618		
*SRSF2* mutations	2.712 (0.627‐11.724)	.182		
Leukemia‐free survival				
*DLX5* methylation	2.744 (1.280‐5.884)	.009	2.394 (1.088‐5.268)	.030
Age	2.014 (1.063‐3.814)	.032	2.061 (1.037‐4.093)	.039
IPSS risks	1.612 (1.113‐2.336)	.012	1.518 (1.039‐2.219)	.031
*CEBPA* mutation	0.044 (0.000‐14.314)	.289		
*IDH1/2* mutation	1.427 (0.193‐10.550)	.728		
*DNMT3A* mutation	2.251 (0.300‐16.877)	.430		
*U2AF1* mutation	1.207 (0.424‐3.436)	.724		
*SF3B1* mutation	0.554 (0.075‐4.063)	.561		
*SRSF2* mutations	2.755 (0.642‐11.826)	.173		

*Note*. Variables including age (≤60 vs > 60 years old), IPSS scores (low vs Int‐1 vs Int‐2 vs high), *DLX5* methylation (nonhypermethylated vs hypermethylated), and gene mutations (mutant vs wild type). Multivariate analysis includes variables with *P *< .100 in univariate analysis.

Abbreviation: IPSS, International Prognostic Scoring System.

### 3.6 *DLX5* exhibited antiproliferative and pro‐apoptotic effects in SKM‐1 cells

**FIGURE 5:**
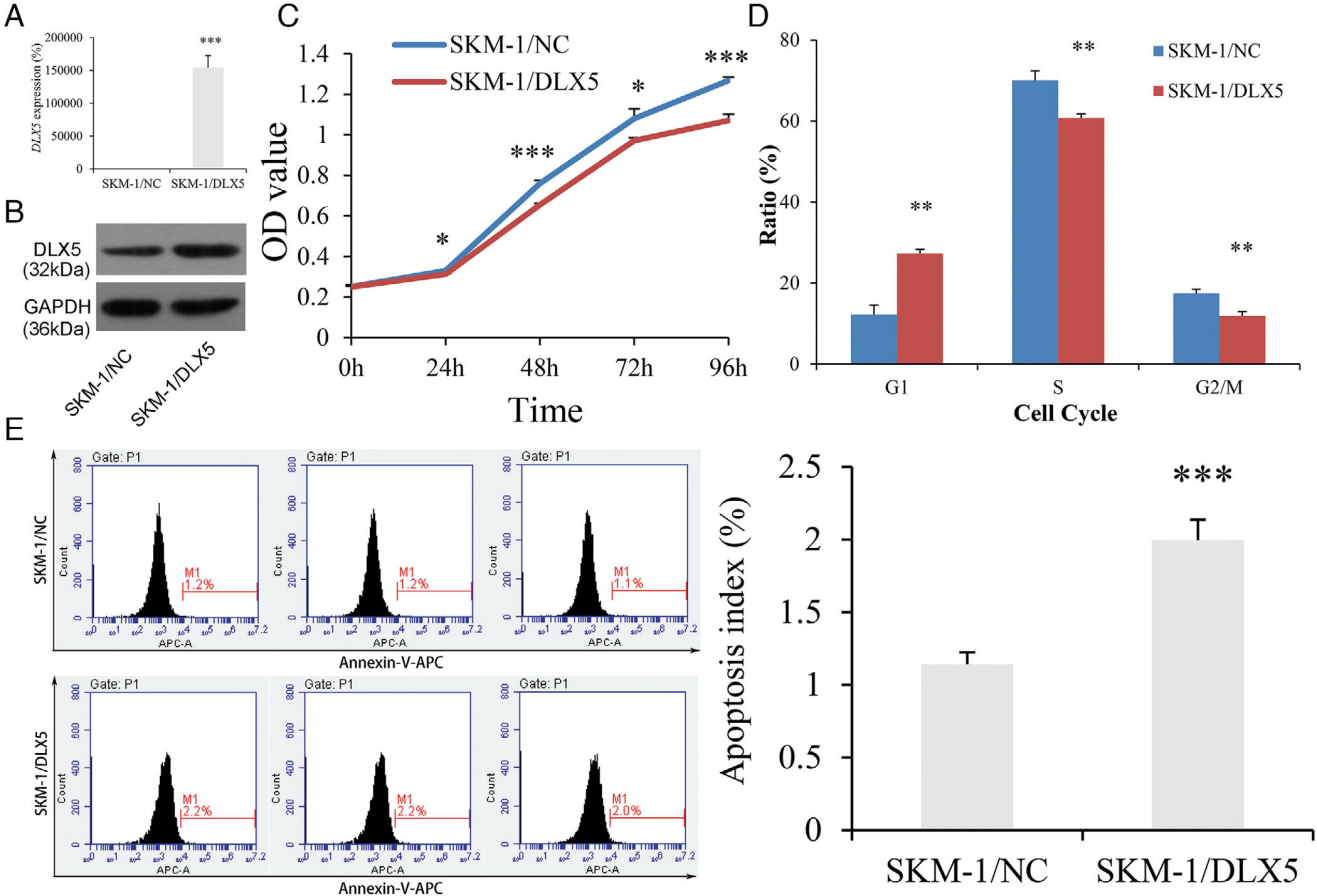
Biological functions of *DLX5* in MDS‐derived AML cell line SKM‐1. A, Confirmation of *DLX5* mRNA level in SKM‐1 after transfection by real‐time quantitative polymerase chain reaction (RT‐qPCR). B, Confirmation of *DLX5* overexpression in SKM‐1 after transfection by western blot. C, The proliferation ability in SKM‐1 affected by *DLX5* overexpression. D, The cell cycle in SKM‐1 affected by *DLX5* overexpression. E, The apoptosis ability in SKM‐1 affected by *DLX5* overexpression. ^*^ *P *< .05; ^**^ *P *< .01; ^***^ *P *< .001

To determine the potential role of *DLX5* in MDS progression, we performed gain‐of‐function experiments in vitro through MDS‐derived AML cell‐line SKM‐1. We established SKM‐1 cells stably overexpressing *DLX5* confirmed by RT‐qPCR and Western blot (Figures 5A and 5B). The proliferation of SKM‐1 cells was markedly inhibited by *DLX5* overexpression (Figure 5C), and caused G0/G1 arrest (Figures 5D and S1). Moreover, we also observed an increased ratio of apoptosis in SKM‐1 cells when *DLX5* overexpressed (Figure 5E).

### 3.7 Molecular insights of *DLX5* in AML

**FIGURE 6:**
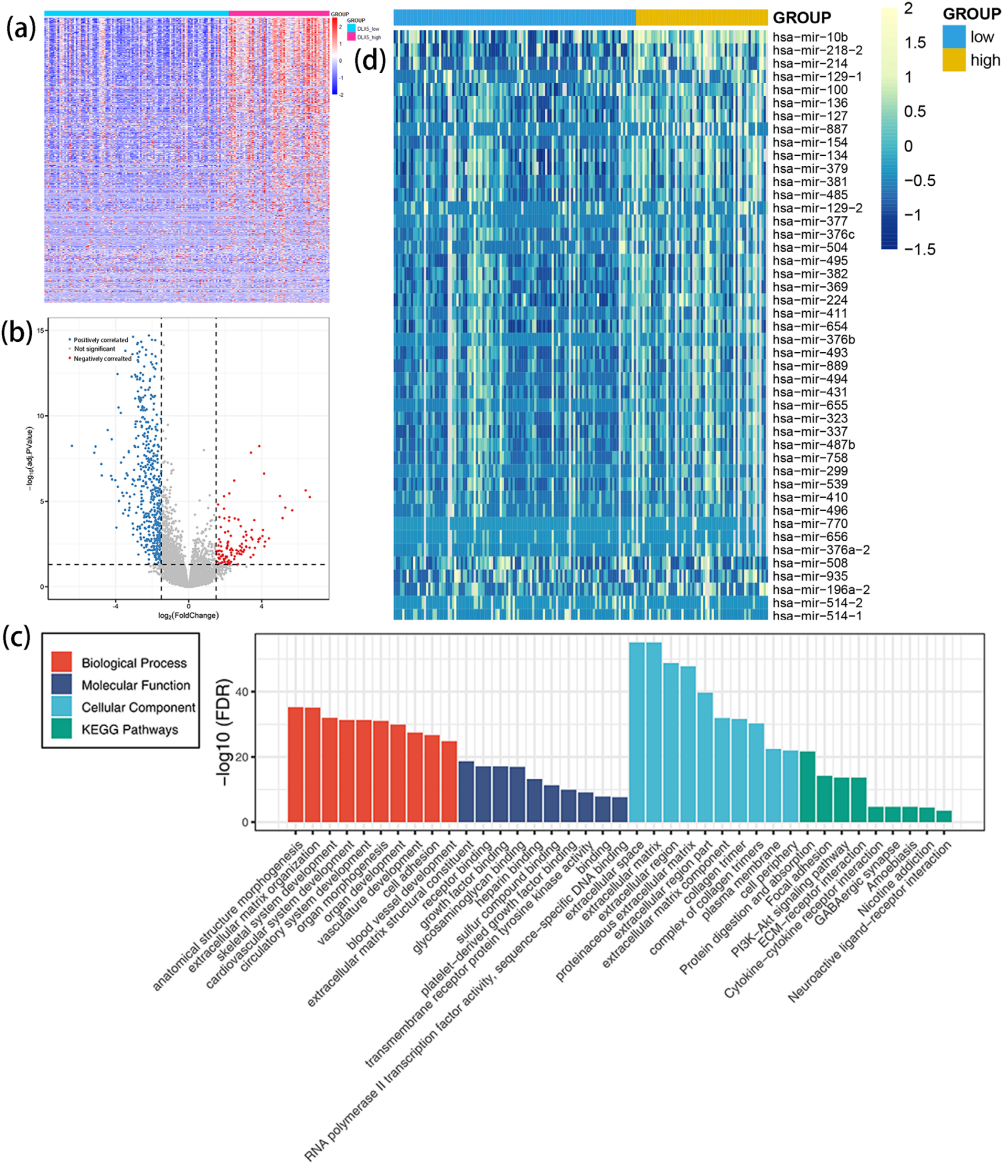
Molecular signatures associated with *DLX5* in AML. A, Expression heatmap of differentially expressed genes between lower and higher expressed *DLX5* in AML patients among TCGA (The Cancer Genome Atlas) datasets (FDR < 0.05, *P *< .05, and |log2 FC| > 1.5). B, Volcano plot of differentially expressed genes between lower and higher expressed *DLX5* in AML patients among TCGA datasets (FDR < 0.05, *P *< .05, and |log2 FC| > 1.5). C, Gene ontology (GO) and Kyoto Encyclopedia of Genes and Genomes (KEGG) analysis of differentially expressed genes by using online website of STRING (http://string-db.org). D, Expression heatmap of differentially expressed microRNAs between lower and higher expressed *DLX5* in AML patients among TCGA datasets (FDR < 0.05, *P *< .05, and |log2 FC| > 1.5)

To gain better understanding of biological insights of *DLX5* in leukemogenesis, we first analyzed the differences of transcriptomes of low‐ and high‐expression *DLX5* groups among 200 AML patients from TCGA databases. We identified 634 differentially expressed genes (FDR < 0.05, *P *< .05, and |log2 FC| > 1.5; Figures 6A and 6B; Supporting Information 1) to be associated with *DLX5* expression. A total of 515 positively correlated genes including *ID4*, *SLIT2/3*, *EBF3*, *WNT5A*, *DKK1*, and *SOX6* were reported with antileukemia effects.^23^, ^29^, ^30^, ^31^ Furthermore, the analysis of gene ontology (GO) and Kyoto Encyclopedia of Genes and Genomes (KEGG) enrichment revealed that these genes were involved in system development, cell adhesion, growth factor binding, and PI3K‐Akt signaling (Figure 6C).

Next, we compared the differences of noncoding small RNA microRNA expression signatures between lower and higher expression *DLX5* groups. We identified 45 differentially expressed microRNAs including 40 positively associated and five negatively associated (FDR < 0.05, *P *< .05, and |log2 FC| > 1.5; Figure 6D; Supporting Information 2). Negatively correlated microRNAs such as *miR‐100*, *miR‐134*, *miR‐504*, *miR‐495*, and *miR‐494* were reported with antileukemia roles in previous works.^32^, ^33^, ^34^, ^35^, ^36^, ^37^

## 4 DISCUSSION

Epigenetic modifications especially in DNA methylation not only involved in leukemogenesis but also served as potential biological markers for AML and MDS patients’ diagnosis and prognosis. Therefore, identifying more aberrant DNA methylation alterations in AML and MDS could make better understanding of leukemogenesis. In the current investigation, we for the first time identified and verified that prognosis‐related *DLX5* expression, screened among all *DLXs* members by public data, was regulated by DNA methylation in AML. By targeted bisulfite sequencing and RT‐qPCR, we further confirmed that *DLX5* hypermethylation was a common event in myeloid neoplasms, and could see as a potential biological marker used in predicting disease prognosis in AML and MDS. Although it is the first time to reveal *DLX5* hypermethylation in myeloid neoplasms, previous study has also proved the phenomena of *DLX5* methylation in breast cancer and neuroblastoma tumors by genome‐wide methylation analysis.^16^, ^17^ All the results suggested the crucial role of the DNA methylation‐mediated *DLX5* silencing during oncogenesis. However, both AML and MDS are heterogeneous diseases, and *DLX5* methylation pattern were not analyzed in single tumor cell population such as CD34+ cell population, leading to a huge difference of *DLX5* methylation in patients with AML and MDS. Accordingly, further studies are required to validate our results before we can use the potential biological markers for risk stratification and planning therapy in AML and MDS.

With regard to prognosis, another important finding in this study was that *DLX5* methylation was associated with MDS progression. Substantial progresses have been made in the understanding of the potential mechanism underlying MDS progression. Genetic alterations especially in gene mutations played vital roles in the disease progression of MDS.^38^, ^39^ Mutations in *TP53*, *TET2*, *IDH1*, *IDH2*, *DNMT3A*, *EZH2*, *ASXL1*, and *ROBO1/2* were considered as progression‐related drivers in MDS.^29^, ^38^, ^39^ Recently, epigenetic changes especially in DNA methylation also have been discovered to be involved in disease progression of MDS.^40^, ^41^, ^42^ Jiang et al reported that abnormal DNA methylation, more frequently occurred than cytogenetic abnormalities, was the major mechanism for silencing tumor suppressor genes and clonal variation in MDS evolution to AML.^42^ Moreover, our study and other investigators also showed that *CDKN2B*, *SOCS1*, *NR4A2*, *ABAT*, *ID4*, *GPX3*, and *SOX30* were associated with MDS progression by signal gene analysis.^21^, ^23^, ^43^, ^44^, ^45^, ^46^, ^47^ These results further give insights in the understanding of the epigenomes of MDS during disease progression, and may provide a theoretical basis for using the efficacy of DNMT inhibitors in MDS patients against disease progression.

In accordance with the clinical studies, the functional studies in vitro showed the antileukemia effects of *DLX5* by affecting cell proliferation and cell apoptosis in MDS‐derived AML cell‐line SKM‐1. Bioinformatics analysis demonstrated that *DLX5* functioned in leukemogenesis may be through the association with PI3K/Akt signaling pathway. However, several studies indicated the oncogenic role of *DLX5* in other human cancers. Tan et al demonstrated that *DLX5* overexpression promoted cell proliferation by enhancing IRS‐2‐AKT signaling in ovarian cancer.^48^ In T‐cell lymphomas, Xu et al reported that *DLX5* could induce tumor cell proliferation by upregulating *MYC* by directly binding to the *MYC* promoter.^49^ Also, Tan et al showed that *DLX5* by directly transactivating Notch signaling and upregulating Akt signaling derived murine T‐cell lymphomagenesis.^50^ All these results suggested that the function of *DLX5* may be specific in different cancer types, and further studies are required to evaluate the biological functions in diverse human cancers.

## 5 CONCLUSION

In summary, our findings demonstrated that *DLX5* methylation, negatively correlated *DLX5* expression, was a potential prognostic and predictive indicator in patients with AML and MDS, which could also act as an epigenetic driver in myeloid neoplasms.

## ETHICAL APPROVAL

The present study was approved by the Ethics Committee and Institutional Review Board of the Affiliated People's Hospital of Jiangsu University. Written informed consents were obtained from all enrolled individuals prior to their participation.

## CONFLICT OF INTEREST

The authors declare no conflict of interest.

## FUNDING

The work was supported by National Natural Science Foundation of China (81900166, 81900163, 81970118, and 81970156), Medical Innovation Team of Jiangsu Province (CXTDB2017002), Natural Science Foundation of Jiangsu Province for Youths (BK20180280), Zhenjiang Clinical Research Center of Hematology (SS2018009), Social Development Foundation of Zhenjiang (SH2017040, SH2018044, SH2019065, and SH2019067), Scientific Research Project of The Fifth 169 Project of Zhenjiang (21), and Youth Medical Talents Project of “Ke Jiao Qiang Wei” Project of Jiangsu Province (QNRC2016450).

## AUTHOR CONTRIBUTIONS

Jing‐dong Zhou and Jun Qian conceived and designed the experiments. Ting‐juan Zhang and Yu Gu performed the experiments. Jing‐dong Zhou and Zi‐jun Xu analyzed the data. Wei Zhang collected the clinical data. Jun Qian, Jing‐dong Zhou, Jiang Lin, Ji‐chun Ma, Xiang‐mei Wen, Zhao‐qun Deng, Wei Zhang, and Jia‐yan Leng offered the technical and funding support. Jing‐dong Zhou wrote the paper. All authors read and approved the final manuscript.

## Supporting information

Supporting Information

Supporting Information

Supporting Information

Supporting Information

## Data Availability

The datasets used and/or analyzed during the current study are available from the corresponding author on reasonable request.
