# Supplementary material for: Identification and validation of prognosis‐related DLX5 methylation as an epigenetic driver in myeloid neoplasms
Source: Clin Transl Med. 2020 Jun 4;10(2):e29. doi: 10.1002/ctm2.29 (PMC7403826; doi:10.1002/ctm2.29)
Supplement: Supplementary file 1 — Supporting Information [file CTM2-10-e29-s001.docx]

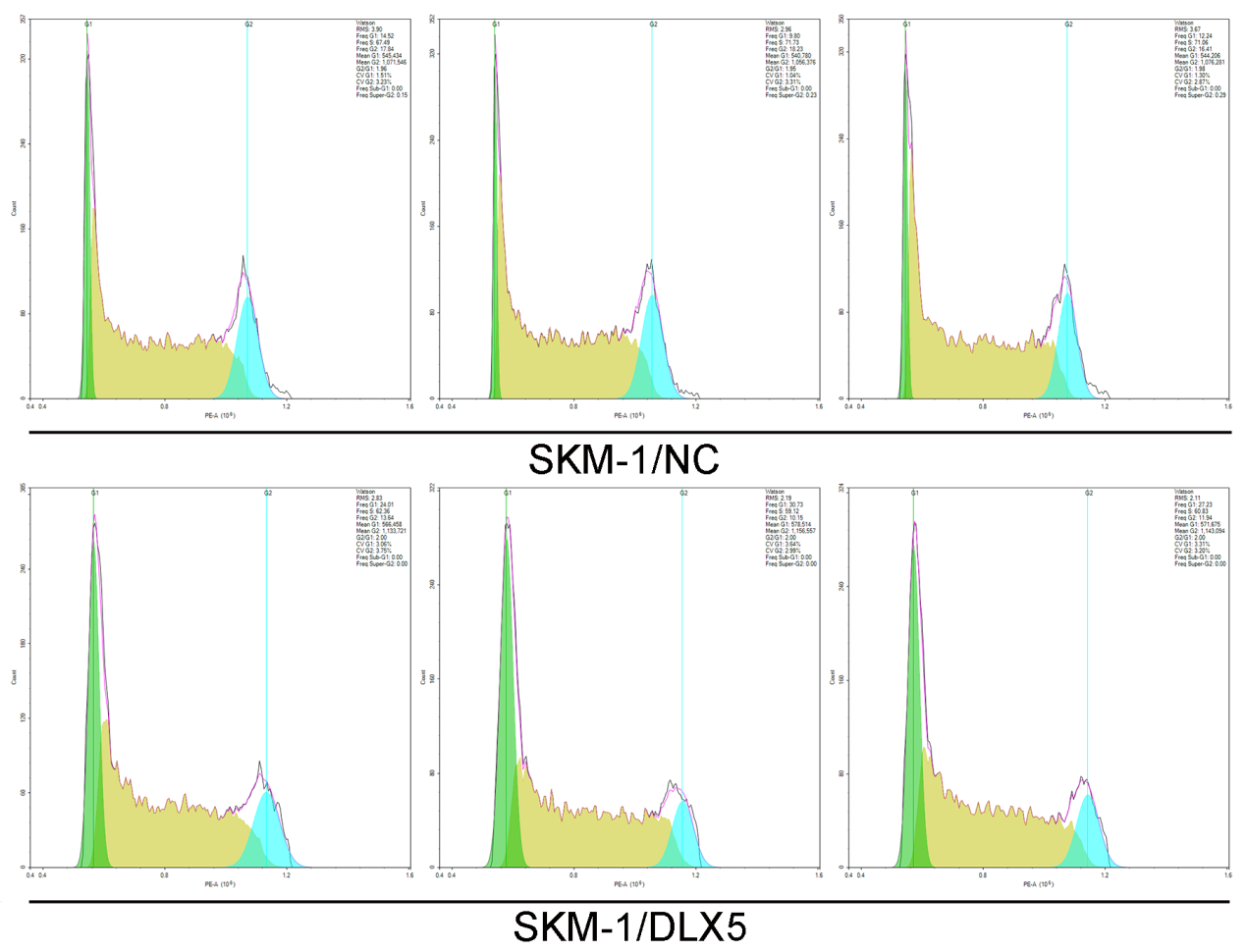


Supplementary material Figure S1. The FACS histogram plot of cell cycle in SKM-1 affected by DLX5 overexpression.
