# Supplementary material for: Identification and validation of prognosis‐related DLX5 methylation as an epigenetic driver in myeloid neoplasms
Source: Clin Transl Med. 2020 Jun 4;10(2):e29. doi: 10.1002/ctm2.29 (PMC7403826; doi:10.1002/ctm2.29)
Supplement: Supplementary file 2 — Supporting Information [file CTM2-10-e29-s004.docx]

**Supplementary material Table S1. Primers used for MethylTarget sequencing, RQ-PCR, and RQ-MSP.**

| Primers | Primer sequence (5’to 3’) |
| --- | --- |
| MethylTarget sequencing |  |
| *DLX5-*F | GGGAGTTTYGTAYGGTTATTGTTTTT |
| *DLX5*-R | CTACCCAACCTTCCCCCTATC |
|  |  |
| RQ-PCR |  |
| *DLX5-*F | CTACCTCGGCTTCCTATGGC |
| *DLX5*-R | TCGGGCTCGGTCACTTCT |
| *ABL1-*F | TCCTCCAGCTGTTATCTGGAAGA |
| *ABL1*-R | TCCAACGAGCGGCTTCAC |
|  |  |
| RQ-MSP primers |  |
| *DLX5*-MF | TTTATTATTAGTACGGCGGC |
| *DLX5*-MR | CGTCCTAATCGCCCTATAT |
| *DLX5*-UF | GTTTTTATTATTAGTATGGTGGT |
| *DLX5*-UR | ACATCCTAATCACCCTATATCT |
| *ALU*-F | TTAGGTATAGTGGTTTATATTTGTAATTTTAGTA |
| *ALU*-R | ATTAACTAAACTAATCTTAAACTCCTAACCTCA |

RQ-PCR: real-time quantitative PCR; RQ-MSP: real-time quantitative methylation-specific PCR

**Supplementary material Table S2. Clinic-pathologic characteristics of four paired MDS/sAML patients used for RRBS**

| Patients | Age/Sex | MDS stage | | | | Progression time (months) | sAML stage | | | |
| --- | --- | --- | --- | --- | --- | --- | --- | --- | --- | --- |
|  |  | WBC/HB/PLT | Blasts | Karyotype | Treatment |  | FAB | WBC/HB/PLT | Blasts | Karyotype |
| P1 | 41/Male | 2.49/91/27 | 16.5% | Normal | IAG | 25 | M6 | 6.07/170/172 | 20% | No data |
| P2 | 36/Male | 1.35/116/12 | 12% | Normal | Supportive | 20 | M6 | 0.36/60/10 | 25% | 47,XX,+21 |
| P3 | 69/Female | 3.43/65/38 | 11% | Complex | Supportive | 11 | M6 | No data | 20.5% | Normal |
| P4 | 74/Female | 5.02/61/72 | 18% | 47,XX,+8 | CAG/C | 20 | M4a | 0.9/63/3 | 55.3% | No data |
| P5 | 76/Female | 4.3/63/78 | 5% | Normal | Supportive | 5 | M2 | 5.2/43/30 | 31% | Normal |
| P6 | 74/Female | 1.2/62/43 | 2% | i(17q) | Thalidomide | 6 | M2 | 2.2/63/11 | 25.5% | i(17q10) |
| P7 | 86/Male | 2.3/43/31 | 14% | Normal | ATRA | 14 | M2a | 5.0/60/36 | 23% | Normal |
| P8 | 62/Male | 1.4/117/93 | 6% | Normal | DA | 2 | M2a | 0.9/99/37 | 20% | Normal |
| P9 | 67/Male | 1.6/66/130 | 9% | Normal | CAG/MA | 24 | M4 | 45/60/55 | 77% | No data |
| P10 | 59/Male | 8.9/62/70 | 7% | Normal | CHG | 23 | M4a | Missing | 85% | Normal |
| P11 | 56/Male | 2.5/107/79 | 11% | Normal | CHG | 7 | M2b | 2.2/77/50 | 41.5% | No data |

WBC: White blood cells (×10^9^/L); HB: Hemoglobin (g/L); PLT: Platelets (×10^9^/L); C: Cytarabine; IAG: Cytarabine+Idarubicin+G-CSF; CAG: Cytarabine+Aclarithromycin+G-CSF; ATRA: All-trans Retinoid Acid; DA: Daunorubicin+ Cytarabine; MA: Mitoxantrone+ Cytarabine; CHG: Cytarabine+ Homoharringtonine +G-CSF.

**Supplementary material Table S3. Logistic regression analyses of variables for complete remission in AML patients**

| Variables | Univariate analysis | | Multivariate analysis | |
| --- | --- | --- | --- | --- |
|  | odds ratio (95% CI) | *P* value | odds ratio (95% CI) | *P* value |
| *DLX5* methylation | 0.483 (0.246-0.947) | 0.034 | 0.452 (0.194-1.057) | 0.067 |
| Age | 0.150 (0.068-0.331) | 0.000 | 0.190 (0.076-0.478) | 0.000 |
| WBC | 0.371 (0.180-0.768) | 0.008 | 0.726 (0.293-1.795) | 0.488 |
| Cytogenetic risks | 0.328 (0.187-0.576) | 0.000 | 0.439 (0.237-0.814) | 0.009 |
| *CEBPA* mutations | 0.722 (0.154-3.384) | 0.680 |  |  |
| *NPM1* mutations | 1.820 (0.502-6.601) | 0.362 |  |  |
| *FLT3*-ITD mutations | 0.801 (0.230-2.793) | 0.728 |  |  |
| *C-KIT* mutations | 2.596 (0.482-13.978) | 0.267 |  |  |
| *N/K-RAS* mutations | 0.333 (0.084-1.329) | 0.119 | 0.315 (0.067-1.471) | 0.142 |
| *DNMT3A* mutations | 0.722 (0.154-2.284) | 0.680 |  |  |
| *U2AF1* mutations | Undetermined | 0.999 |  |  |
| *IDH1/2* mutations | 0.482 (0.042-5.473) | 0.556 |  |  |
| *SRSF2* mutations | Undetermined | 0.999 |  |  |
| *SETBP1* mutations | 0.982 (0.060-16.097) | 0.990 |  |  |

Variables including *DLX5* methylation (hypermethylation vs. non-hypermethylation), age (≤60 vs. >60 years), WBC (≥30×10^9^ vs. <30×10^9^ /L), and gene mutations (mutant vs. wild-type). Multivariate analysis includes variables with *P*<0.200 in univariate analysis.

**Supplementary material Table S4. Cox regression analyses of variables for overall survival in AML patients**

| Variables | Univariate analysis | | Multivariate analysis | |
| --- | --- | --- | --- | --- |
|  | hazard ratio (95% CI) | *P* value | hazard ratio (95% CI) | *P* value |
| *DLX5* methylation | 1.832 (1.229-2.728) | 0.003 | 1.536 (0.965-2.445) | 0.071 |
| Age | 2.840 (1.911-4.222) | 0.000 | 2.160 (1.371-3.402) | 0.001 |
| WBC | 2.112 (1.423-3.135) | 0.000 | 1.474 (0.940-2.313) | 0.091 |
| Cytogenetic risks | 1.794 (1.426-2.258) | 0.000 | 1.597 (1.170-2.181) | 0.003 |
| *CEBPA* mutations | 1.703 (0.737-3.934) | 0.213 |  |  |
| *NPM1* mutations | 0.704 (0.306-1.621) | 0.410 |  |  |
| *FLT3*-ITD mutations | 0.861 (0.396-1.872) | 0.705 |  |  |
| *C-KIT* mutations | 0.766 (0.280-2.094) | 0.603 |  |  |
| *N/K-RAS* mutations | 1.200 (0.576-2.497) | 0.627 |  |  |
| *DNMT3A* mutations | 1.244 (0.541-2.864) | 0.607 |  |  |
| *U2AF1* mutations | 3.103 (0.954-10.087) | 0.060 | 3.470 (1.058-11.380) | 0.040 |
| *IDH1/2* mutations | 1.311 (0.321-5.356) | 0.706 |  |  |
| *SRSF2* mutations | 2.586 (0.933-7.169) | 0.068 | 1.817 (0.651-5.077) | 0.254 |
| *SETBP1* mutations | 0.778 (0.108-5.601) | 0.803 |  |  |

Variables including *DLX5* methylation (hypermethylation vs. non-hypermethylation), age (≤60 vs. >60 years), WBC (≥30×10^9^ vs. <30×10^9^ /L), and gene mutations (mutant vs. wild-type). Multivariate analysis includes variables with *P*<0.200 in univariate analysis.
